# AI-Enabled Parabolic Response Surface Approach Identifies Ultra Short-Course Near-Universal TB Drug Regimens

**DOI:** 10.1002/adtp.201900086

**Published:** 2019-09-18

**Authors:** Marcus A. Horwitz, Daniel L. Clemens, Bai-Yu Lee

**Keywords:** drug combination optimization, drug regimens, drug synergy, mycobacterium tuberculosis, tuberculosis

## Abstract

Tuberculosis (TB) is a major health problem that causes more deaths worldwide than any other single infectious disease. Current multidrug therapy for tuberculosis is exceedingly lengthy, leading to poor drug adherence, and consequently the emergence of drug resistance. Hence, much more rapid treatments are needed. Experimentally identifying the most synergistic drug combinations among available drugs is complicated by the astronomical number of possible drug-dose combinations. This problem is dealt with by the use of an artificial-intelligence-enabled parabolic response surface platform in conjunction with an in vitro *Mycobacterium tuberculosis*–infected macrophage cell culture assay amenable to high-throughput screening. This strategy allows rapid identification of the most effective drug-dose combinations by testing only a small fraction of the total drug-dose efficacy response surface. The same platform is then used to optimize the in vivo doses of each drug in the most potent regimens. Thus, regimens are identified that are dramatically more effective than the Standard Regimen in treating TB in a mouse model—a model broadly predictive of drug efficacy in humans. The most effective regimens reported herein shorten the duration of treatment required to achieve relapse-free cure by 80% and are suitable for treating both drug-sensitive and most drug-resistant cases of tuberculosis.

## 1. Introduction

Tuberculosis (TB) remains one of the world’s greatest health problems. While the incidence of TB is low in “First World” countries, for example, Western European countries and the United States, the disease continues to ravage people in less developed areas of the world, especially in Asia, Africa, and Latin America. TB is the leading cause of death from a single infectious agent, killing more people than AIDS and malaria. According to WHO, in 2017, the latest year for which data are available, ≈10.0 million people developed TB disease and 1.6 million people died, including 300 000 people co-infected with HIV.^[1]^ Approximately 1.7 billion people are infected with *Mycobacterium tuberculosis* (Mtb), the causative agent, of whom ≈10% will develop active disease some time during their lifetime.^[1]^ People with HIV or immunocompromised immunity are more susceptible to developing TB; other risk factors include malnutrition, diabetes, alcoholism, and smoking. Complicating the TB pandemic is the emergence of strains of Mtb resistant to the major drugs used to treat TB.

Treatment for TB is unusually onerous, requiring prolonged therapy with a multidrug regimen to prevent the emergence of drug resistance. For patients with drug-sensitive TB, treatment typically requires 6–8 months but can sometimes require much longer—even 18 months. For patients with drug-resistant TB, treatment typically requires a minimum of 9–12 months and as long as 24 months. The standard treatment for drug-sensitive TB is a four-drug regimen (Standard Regimen) comprising isoniazid (INH), rifampin (RIF), ethambutol (EMB), and pyrazinamide (PZA). Patients with multidrug resistant TB (MDRTB) are by definition infected with Mtb resistant to at least INH and RIF, the two most important drugs in the Standard Regimen. Even worse, patients with extensively drug-resistant TB (XDRTB) are not only resistant to INH and RIF but also to aminoglycosides and fluoroquinolones, two other major classes of drugs commonly used to treat TB.

The long treatment course for TB provokes nonadherence to the drug regimen, and this in turn leads to the emergence of drug-resistant TB, for which treatment has a lower success rate. Worldwide, the treatment success rate for drug-sensitive TB is *>*85%; for MDRTB, the success rate drops to ≈54%.^[1]^ Hence, much faster and more potent drug regimens are needed to combat the TB pandemic.

To identify highly synergistic drug combinations to treat TB, we have employed the artificial-intelligence-enabled parabolic response surface (AI-PRS) approach. The AI-PRS approach is a new paradigm for identifying highly synergistic drug regimens for the treatment of disease based upon the observation that drug-dose inputs are correlated with phenotypic outputs (e.g., amelioration of a disease state) in numerous biological systems by a parabolic response surface; such a surface is described by a quadratic algebraic equation.^[2–5]^ The AI-PRS approach, which is output driven and hence agnostic to drug mechanism, has been applied to identify synergistic drug combinations to address a large variety of medical problems including treatment of cancer (hepatocellular, breast, ovarian, colon, renal, and bladder carcinoma; multiple myeloma; acute lymphoblastic leukemia) and infectious diseases (herpes simplex virus-1 infection; parasitic nematode infection) and suppression of rejection in liver transplantation.^[2–13]^

## 2. Evolution of TB Drug Regimens—One Drug Changed at a Time by Trial and Error

The first multidrug TB regimen, a three-drug regimen, was developed in 1952 and required 24 months of therapy to achieve cure.^[14]^ Over the next 30 years, this regimen was changed and improved several times by substituting or adding one drug at a time until the current Standard Regimen was attained in the 1980s, a regimen typically requiring 6–8 months of therapy. Synergy was not a major consideration in the development of these regimens. Indeed, directly contrary to the concept of synergy, two of the four drugs in the Standard Regimen (EMB and PZA) are dropped after 2 months therapy. Typically, the drugs used in TB drug regimens have been administered at the maximum well-tolerated dose, which, because of drug–drug interactions, is not always the optimal dose.

## 3. The Drug-Dose Dilemma

Identifying highly synergistic drug combinations is complicated by the drug-dose dilemma that arises because the optimal doses of drugs used in combination are often well below the maximum well-tolerated dose. Testing all possible drug and dose combinations to determine optimal drug dosage ratios for even a small number of drugs and doses is practically impossible. For *N* drugs at *M* dosage levels, the number of possible combinations is *M^N^*. Thus, for example, testing 14 TB drugs at just five dosage levels would require testing 5^14^ combinations or ≈6.2 billion combinations. The AI-PRS approach provides a solution to this dilemma.

## 4. AI-PRS Approach to Identify Synergistic TB Drug Regimens

We have applied AI-PRS technology with substantial success to identify highly synergistic drug regimens with which to treat TB.^[15–18]^ Our most successful regimens, described below, allow the treatment time for TB to be reduced by 80% compared with the Standard Regimen in the BALB/c mouse model of pulmonary TB, a model highly predictive of drug efficacy in humans.

The basic premise of the PRS approach, based upon numerous in vivo observations of drug effects on a variety of biological conditions, is that the efficacy of drugs at different doses is described with reasonable accuracy by a smooth parabolic surface, that is, there are no abrupt changes in efficacy with small changes in dose. Such a surface is described by a second-order algebraic equation: Equation (1)

$$\begin{array}{l} y=\beta_{0}+\beta_{1}x_{1}+...+\beta_{n}x_{n}+\beta_{12}x_{1}x_{2}+...+\beta_{mn}x_{m}x_{n}+\beta_{11}x_{1}^{2}+\beta_{nm}x_{n}^{2} \end{array}$$ 1

Optimal drug-dose combinations can be identified and the PRS surface mapped by solving this equation, which requires testing only a relatively few drug-dose combinations. For example, we identified highly synergistic drug combinations for treating TB from among 14 different TB drugs by performing a few hundred tests (i.e., several iterations of ≈100 tests per iteration) as described below. Importantly, the PRS approach is output driven, in this case based upon the efficacy of TB drugs in killing Mtb, and hence it is agnostic to such considerations as drug mechanism, pharmacokinetics, etc. The approach informs as to what is efficacious but does not explain why or how.

### 4.1. PRS Approach in Practice

**Figure 1:**
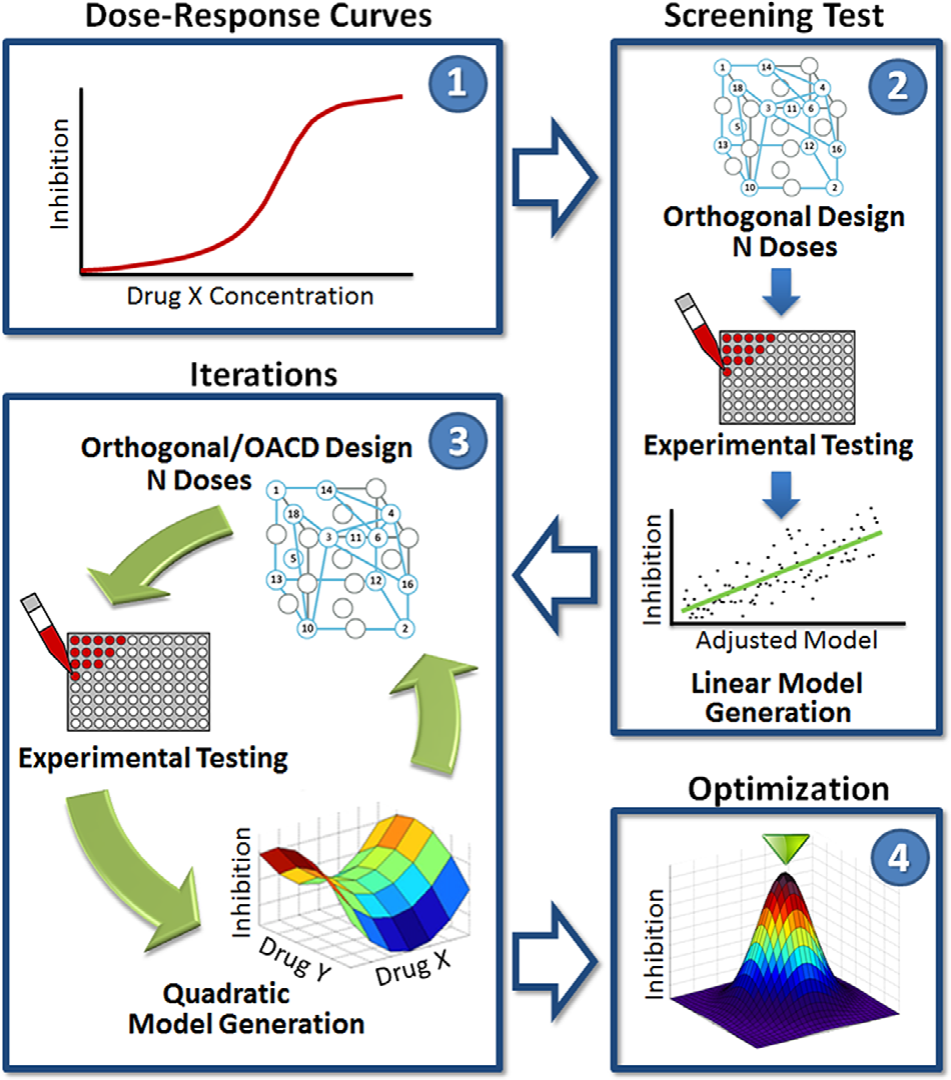
PRS schematic. The diagram shows the four phases of PRS methodology employed to identify highly potent drug combinations at their optimal doses using the fluorescence-based Mtb-infected macrophage assay. The four phases are: 1) a dose-response curve is established for each drug; 2) a screening test is carried out using a two-level (two doses) orthogonal array design; the results are used to construct a first-order linear model; 3) multiple iterations based on an orthogonal or orthogonal array central composite design (OACD) are carried out to evaluate drug combinations; the results are used to construct a second-order quadratic model and a surface model; and 4) optimization of the drug combinations and the drug ratios from the final surface model that was constructed in Phase 3. Reproduced with permission.^[15]^ Copyright 2016, U.S. National Academy of Sciences.

In practice, several steps are involved (**Figure 1**). First, one establishes an accurate dose-response curve for each drug—in this case, the capacity of a drug to inhibit Mtb, so that one can add doses of different drugs at comparable inhibitory levels, for example, the 10%, 15%, or 20% inhibitory level. Second, in a screening test, one evaluates just two doses of each drug (e.g., 0% and 10%) in combination with other drugs to generate a linear model of efficacy described by a first-order algebraic equation. Regression analysis allows elimination of drugs that are antagonistic or clearly do not contribute to efficacy. Third, one tests in several iterations of about 100 tests each, first three, then four, and then five doses of each drug in combination with the other drugs to generate an increasingly accurate quadratic model. Ultimately, one obtains the optimized drug combinations from the final surface model. This provides a rank order of all possible drug combinations at their optimized drug ratios, for example, a rank order of the 1001 four-drug combinations among 14 TB drugs.^[15]^

### 4.2. The Critical Choice of In Vitro Assay

The identification of synergistic drug regimens via the PRS approach, while requiring fewer tests than traditional approaches to identify synergistic drug combinations, nevertheless requires a substantial number of tests such that, practically speaking, an in vitro model is necessary. Ideally, the model should be amenable to high-throughput screening. In any case, for the approach to be successful, it is critical that the in vitro model reflect the in vivo situation. In the case of TB, we employed as our in vitro model, Mtb infection of human macrophages, the host cells for Mtb in vivo. Typically, TB drug studies are carried out with Mtb grown in broth culture under various conditions; however, such conditions are a poor reflection of the in vivo situation where Mtb multiplies extensively within macrophages. That our Mtb-infected macrophage model successfully predicted drug combinations with high efficacy in vivo in a mouse model of pulmonary TB is a testament to the utility of this particular in vitro model.

**Figure 2:**
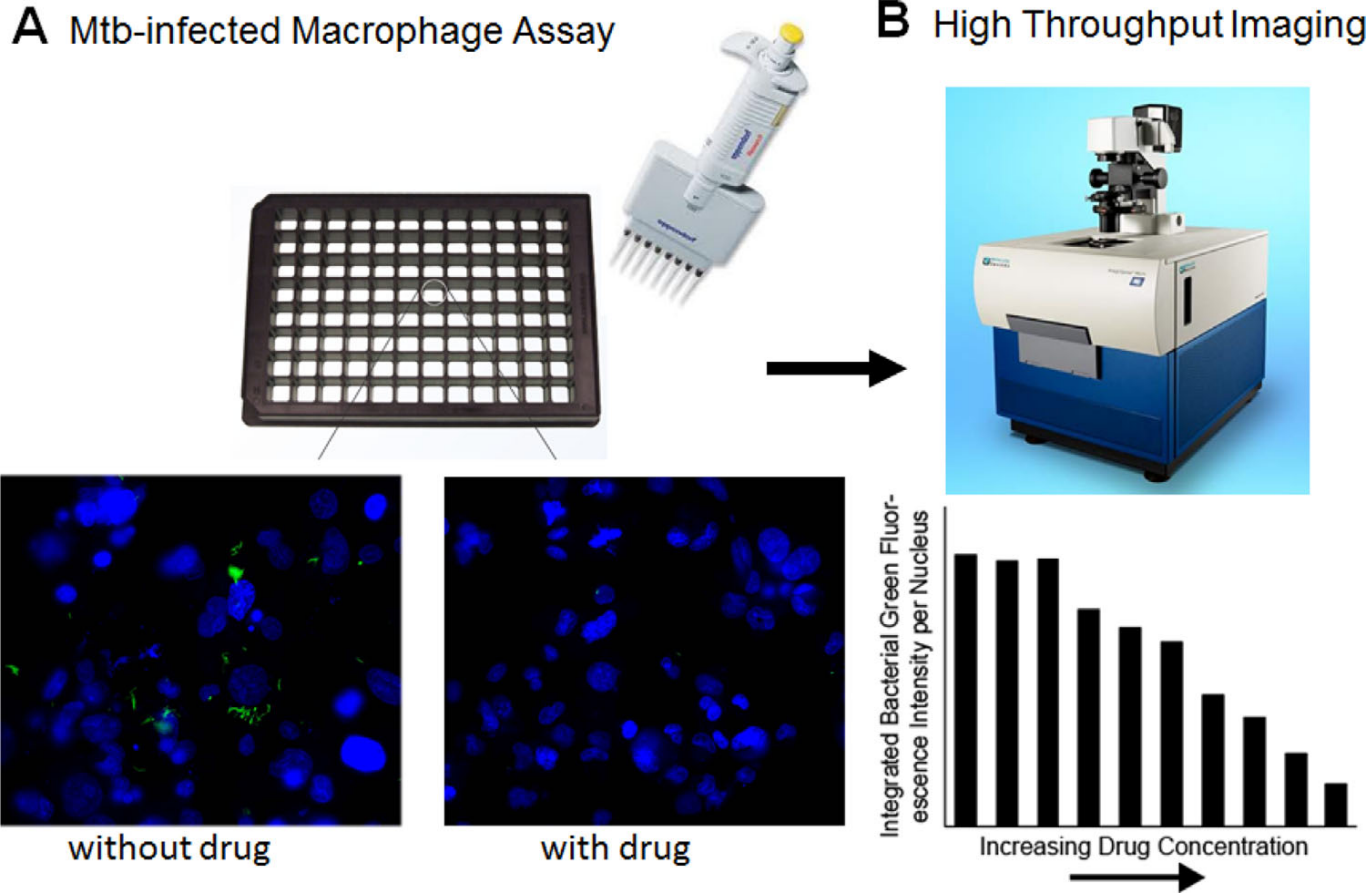
PRS screening assay for drug inhibition of Mtb. We employed an assay compatible with high-throughput screening to assess drug-dose combinations. A) Mtb-infected macrophage assay. Macrophages are infected in 96-well plates with Mtb engineered for IPTG-inducible green fluorescence (upper images). Then drugs at various drug-dose combinations and IPTG are added to the wells, and the culture is incubated for 4 days and imaged. Under a fluorescent microscope, with no drug present, green fluorescent bacteria are readily apparent (lower left image)—the blue fluorescent structures are the nuclei of macrophages stained with Hoechst dye. In contrast, in the presence of a drug that kills the bacteria, the green fluorescent bacteria are absent (lower right image). B) High-throughput imaging. The plates are read with a high-throughput machine that calculates the integrated green fluorescence intensity per macrophage nucleus (upper image). A typical dose-response to a drug active against Mtb is shown in the lower image. With increasing drug concentration, the green fluorescence and thus the number of live bacteria per macrophage nucleus decreases.

In our high-throughput screening system, we infect macrophages with Mtb in 96-well plates and then add various antibiotic combinations (**Figure 2**A, upper panel). To facilitate high-throughput screening of the viability of Mtb in the infected macrophages after antibiotic treatment, we employ a highly virulent Mtb Erdman strain engineered for isopropyl *β*-D-1-thiogalactopyranoside (IPTG)-inducible green fluorescence developed for this purpose in our laboratory.^[19]^ Since IPTG-inducible fluorescence requires bacterial metabolism, in the presence of an antibiotic capable of killing Mtb, fluorescence is abolished (Figure 2A, lower panel). After adding antibiotics and IPTG, we incubate the infected macrophages for 4 days, fix the cells, treat them with Hoechst dye, which stains the macrophage nuclei blue, and then use an automated epifluorescence microscope and imaging analysis software to quantitate the integrated bacterial green fluorescence intensity per blue macrophage nucleus^[15]^ (Figure 2B). The use of an Mtb strain requiring IPTG induction for fluorescence results in very low background fluorescence in our high-throughput screening assay. A typical dose-response curve for a single antibiotic is shown in Figure 2B, lower panel.

## 5. PRS Regimens I and II Markedly Shorten TB Treatment Time

**Figure 3:**
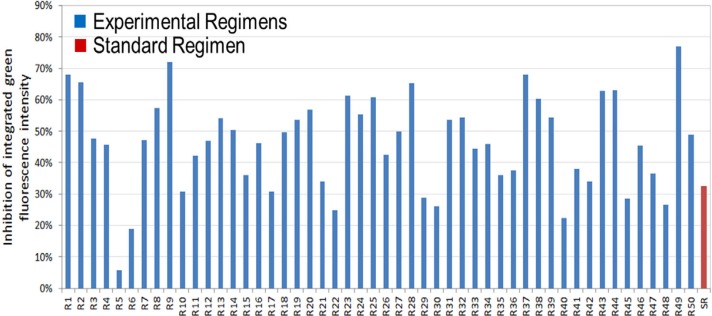
The PRS approach identifies numerous TB drug regimens that are more effective than the Standard Regimen in inhibiting Mtb growth in macrophages. The graph shows the extent of inhibition of integrated mean fluorescence intensity of the first 50 experimental regimens and of the Standard Regimen from part of a run of a PRS in vitro study.^[15]^ The experimental regimens are shown in blue and the Standard Regimen is shown in red. Many of the experimental regimens are more effective than the Standard Regimen in killing Mtb in macrophages.

In our first series of studies,^[15,16]^ we sought highly synergistic TB drug combinations among 14 TB drugs suitable for oral administration including the four first line TB drugs comprising the Standard Regimen (INH, RIF, EMB, and PZA); the second line TB drugs moxifloxacin(MXF), para-aminosalicyclic acid (PAS), prothionamide (PRO), cycloserine (CYS), and bedaquiline (BDQ); the third line drugs amoxicillin/clavulanate (A/C), clofazimine (CFZ), and linezolid (LZD); and the experimental drugs PA-824 (pretomanid) and SQ109. The Standard Regimen was studied as a control. We identified numerous TB drug regimens that weremore efficacious than the Standard Regimen (**Figure 3**). The efficacy of the most potent regimens was verified in an orthogonal in vitro macrophage killing assay in which Mtb viability was assessed by assaying colony-forming units (CFU) of Mtb after treatment with the drug regimens. Among the top regimens, we initially selected the most potent generic drug regimen (PRS Regimen I), comprising CFZ, EMB, PRO, and PZA, and the most potent regimen (PRS Regimen II) comprising approved drugs (CFZ, EMB, BDQ, and PZA) for further study in vivo.

### 5.1. In Vivo Evaluation of PRS Regimens I and II

We next sought to determine if the highly potent PRS regimens identified in in vitro studies had greater efficacy than the Standard Regimen in vivo.^[16]^ For this evaluation, we used a murine model of pulmonary tuberculosis, which has typically been broadly predictive of the efficacy of TB drug regimens in humans. In this model, BALB/c mice are infected by aerosol with ≈100 CFU of the highly virulent Mtb Erdman strain and then rested for 2 weeks, during which time Mtb multiplies in their lungs by several logs to ≈6 or 7 log CFU. The mice are then sham-treated or treated with the various drug regimens by gavage and the lung burden assayed after various treatment durations.

### 5.2. Optimizing In Vivo Drug Doses by Mapping the Drug-Dose Efficacy Response Surface

Evaluating the PRS regimens in vivo first requires optimizing the in vivo doses of the drugs in each regimen. This is of critical importance because the optimal in vitro and in vivo drug ratios may differ substantially, for example, because drug absorption, metabolism, and distribution have a very significant impact on the availability of drugs in vivo. Extrapolating drug doses from the in vitro to the in vivo situation is conventionally accomplished using pharmacokinetic data and drug scaling; however, this approach is highly problematic. Instead, we use the output-driven PRS approach to optimize the in vivo drug doses as this approach is agnostic to such considerations as drug mechanism, the metabolic state of the bacteria, and drug pharmacokinetics, and automatically takes into account drug–drug interactions. As with in vitro applications of the PRS approach, the relationship between the phenotypic response and the drug doses in vivo fits a parabolic response surface, which we refer to as the drug-dose efficacy response surface. This surface, as noted above, can be described by a second-order algebraic equation and a relatively small number of tests are required to accurately map it. Our drug combinations contain four drugs, and ordinarily, 15 tests would be required to solve the 15 coefficients of the second-order algebraic equation for four drugs. However, in our studies, we chose to hold the dose of one of the drugs (CFZ) constant because of its extraordinarily long half-life and atypical pharmacokinetics. Hence, we required only ten tests to solve the ten coefficients of the second-order algebraic equation for three drugs. Thus, using the PRS approach, we were able to transition drug doses from the in vitro to in vivo situation time- and cost-effectively.

**Figure 4:**
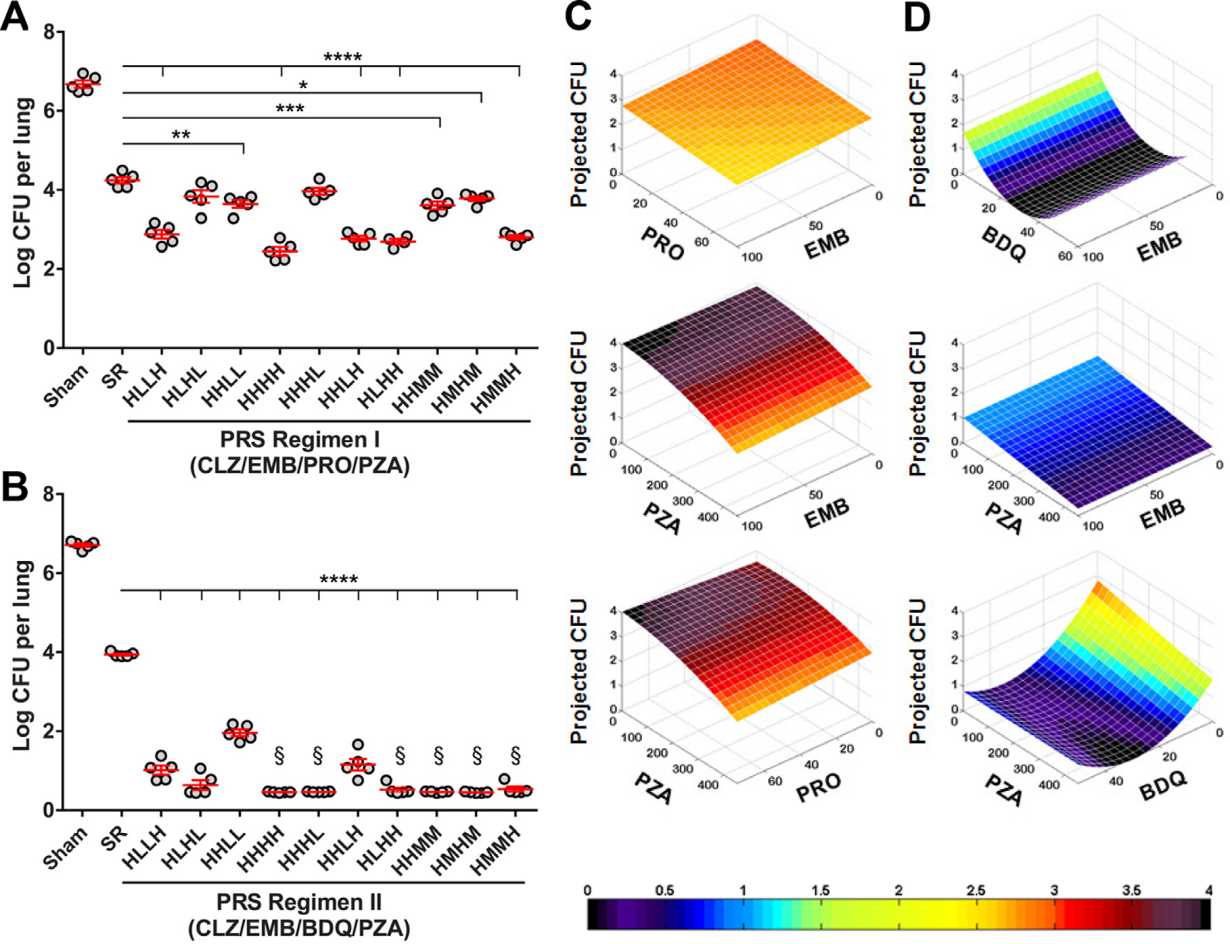
In vivo optimization of drug doses in PRS Regimens I and II. Lung burden of Mtb in mice that were sham-treated, treated with the Standard Regimen (SR), or treated with A) PRS Regimen I or B) PRS Regimen II with the drugs administered at high (H), middle (M), or low (L) dose five times per week for 4 weeks. CFZ was kept constant at the high dose. Data are mean ± SEM of log10 CFU for *n* = 5 mice per group. All treatment groups had significantly fewer CFU than the sham-treated group (*p <* 0.0001). Differences in treatment efficacy between the Standard Regimen and individual PRS Regimen I or II groups were evaluated by one-way ANOVA. *p < 0.05, **p < 0.01, ***p < 0.001, ****p < 0.0001. §No Mtb CFU detected—data were plotted at ½ the limit of detection. Heat maps of the drug-dose efficacy response surface for C) PRS Regimen I and D) PRS Regimen II. These 3D graphs show how the projected number of lung log CFU changes as the dose of one and/or the other drug is increased or decreased. Drug dose is shown in mg kg^−1^. In these plots, in addition to CFZ, the third drug is kept at the high dose. Reproduced with permission.^[16]^ Copyright 2017, Springer Nature.

In our mapping studies, drugs are administered at a high dose equal to the highest well-tolerated dose, a medium dose equal to one-third the high dose, and a low dose equal to one-ninth the high dose. Drugs are administered 5 days per week (Monday– Friday) by gavage over a 4-week period, a time period insufficiently long for the best drug dose combinations to sterilize the lungs in the cases of PRS Regimens I and II, thus allowing meaningful comparisons of efficacy among the different groups of mice. The results of the mapping studies of PRS Regimens I and II are shown in **Figure 4**. Of note, all drug-dose combinations were better than the Standard Regimen, used for comparison, and the best groups were substantially better. For example, while the Standard Regimen was somewhat effective, reducing the lung burden by ≈2.5 logs in comparison with sham-treated mice in each study, the best drug dose ratios in the case of PRS Regimen I reduced the lung burden by an additional 1.8 logs compared with the Standard Regimen and the best drug dose ratios in the case of PRS Regimen II reduced the lung burden by an additional 3.2 logs compared with the Standard Regimen. The optimal drug doses for CFZ, EMB, PRO, and PZA in PRS Regimen I were determined to be 25, 100, 75, and 450 mg kg^−1^, respectively, and for CFZ, EMB, BDQ, and PZA in PRS Regimen II to be 25, 100, 30, and 450 mg kg^−1^, respectively. The drug doses used for the Standard Regimen were 25, 10, 100, and 150 mg kg^−1^ for INH, RIF, EMB, and PZA, respectively; EMB and PZA were dropped after 8 weeks as in the treatment of TB in humans. These doses were originally extrapolated to the mouse from optimized human doses on the basis of pharmacologic data (serum AUC equivalent to that of recommended human doses).^[20,21]^ As they are the conventional doses of the Standard Regimen drugs used in mouse efficacy studies, they serve as an important benchmark for comparisons among TB drug studies.

### 5.3. PRS Regimens I and II Rapidly Sterilize the Lungs and Markedly Shorten the Treatment Time Needed to Achieve Relapse-Free Cure

**Figure 5:**
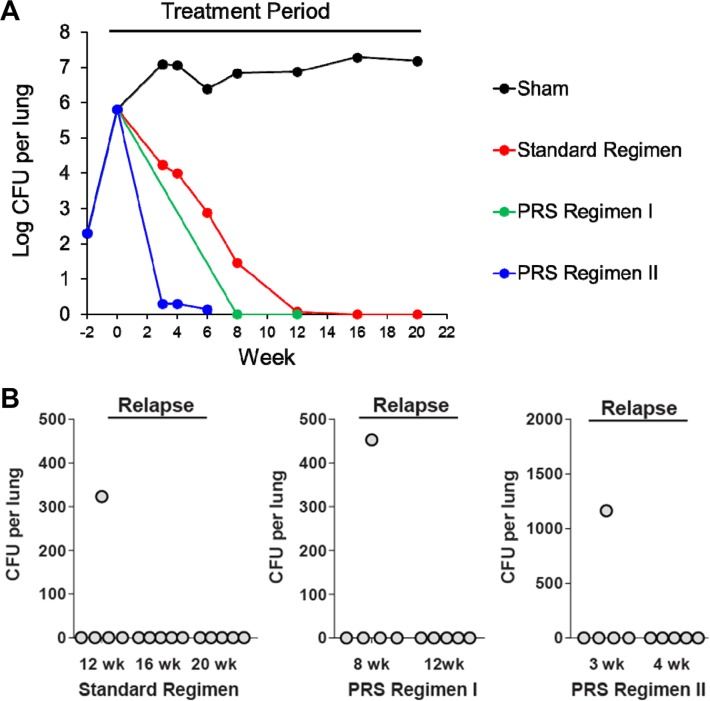
PRS Regimens I and II: long-term efficacy (time to lung sterilization) and relapse (time to relapse-free cure) study. A) Efficacy. Mtb burden in the lung over the course of infection and treatment period, where mice were sham-treated or treated with the Standard Regimen, PRS Regimen I, or PRS Regimen II for 5 days (Monday–Friday) per week starting at Week 0. Data transformation as log10 (*x* + 1) with *x* being the actual number of CFU used for graphing purpose. B) Relapse. Relapse in the lung 3 months after completion of treatment with PRS Regimen I or II or the Standard Regimen 5 days per week for the duration indicated. Differences in time to relapse-free cure between the Standard Regimen and the PRS regimens were statistically significant (*p* = 0.002 vs PRS Regimen I and *p <* 0.0001 vs PRS Regimen II, log rank test). Differences between PRS Regimens I and II in time to relapse-free cure were also statistically significant (*p <* 0.0001, log rank test). Adapted with permission.^[16]^ Copyright 2017, Springer Nature.

**Figure 6:**
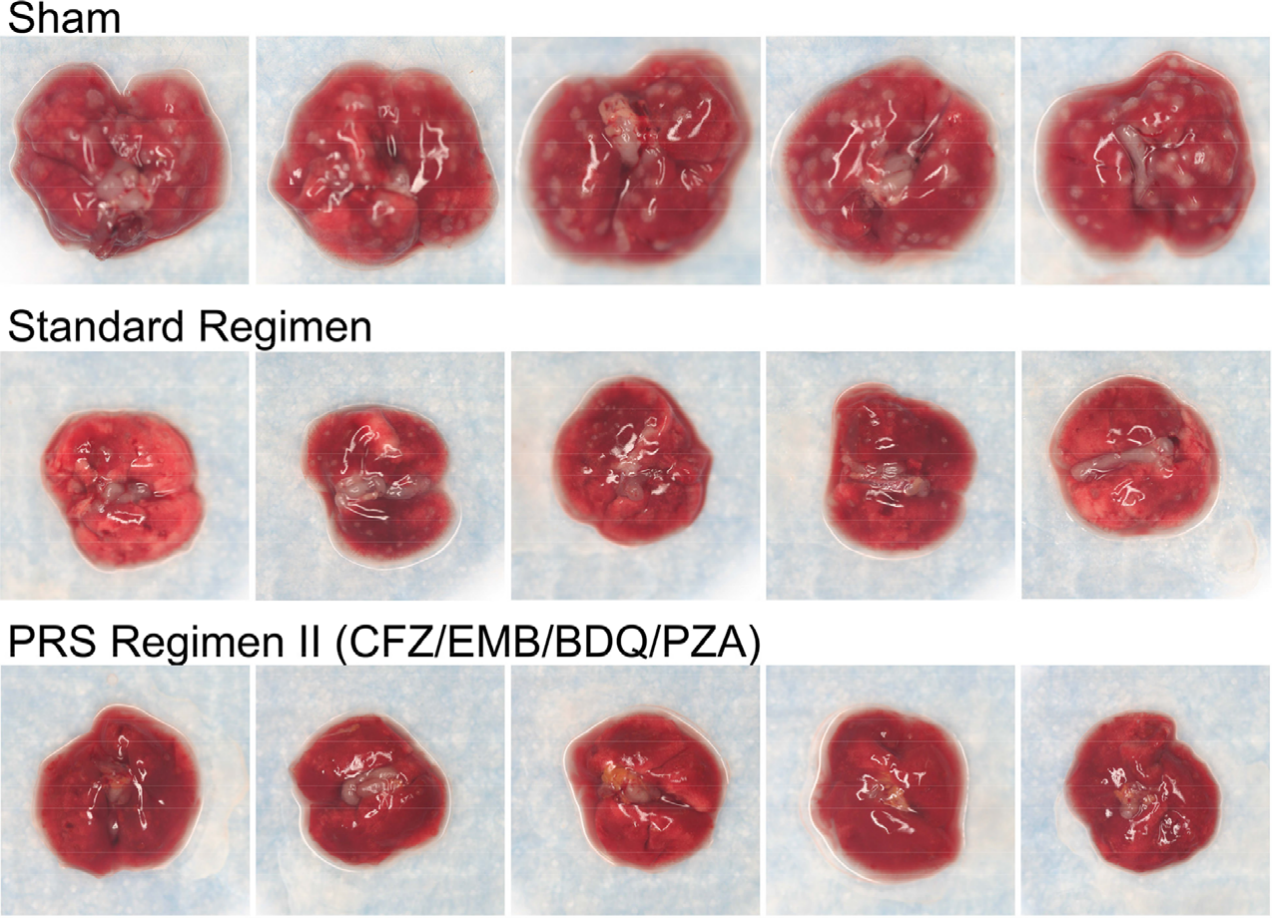
PRS Regimen II: lung pathology. Shown are representative gross pathology images of lungs dissected from BALB/c mice that were sham-treated or treated with the Standard Regimen or PRS Regimen II at high doses of each drug by oral gavage five times (Monday–Friday) per week for 4 weeks. Lungs from sham-treated mice are peppered with large granulomas. Lungs from mice treated with the Standard Regimen have substantially fewer and smaller granulomas. Lungs from mice treated with PRS Regimen II are virtually devoid of granulomas. Reproduced with permission.^[16]^ Copyright 2017, Springer Nature.

After optimizing their in vivo drug doses, PRS Regimens I and II were evaluated for efficacy in the BALB/c mouse model of pulmonary TB. Two types of efficacy studies were conducted. In the first type of study (time to lung sterilization), the regimens were evaluated for the time required to sterilize the lungs by treating groups of mice by gavage (5 days per week) with the regimens for various lengths of time, starting 2 weeks after infection (by which time Mtb has multiplied to a high level); and then euthanizing the mice and culturing the entire lung on agar to determine CFU of Mtb remaining in the lung. In the second type of study (time to relapse-free cure), the mice were treated for various lengths of time as in the time to sterilization study, then left untreated for 3 months, and then euthanized to determine CFU of Mtb in the lungs. The Standard Regimen for treating TB served as a control. Relapse-free cure was defined as the absence of CFU in the entire lung 3 months after cessation of treatment. Compared with the Standard Regimen, PRS Regimens I and II more rapidly sterilized the lungs, and PRS Regimen II reduced CFU much more rapidly than PRS Regimen I (**Figure 5A**). Paralleling the more rapid reduction in CFU, mice treated with the PRS Regimens showed less lung pathology than mice treated with the Standard Regimen (**Figure 6**).

PRS Regimens I and II also achieved relapse-free cure much more rapidly than the Standard Regimen. Whereas the Standard Regimen required 16 weeks to achieve relapse-free cure, in the same experiment, PRS Regimen I required only 12 weeks and PRS Regimen II only 4 weeks. (Figure 5B). Hence, compared with the Standard Regimen, PRS Regimens I and II reduced the time required to obtain relapse-free cure by 25% and 75%, respectively.

## 6. PRS Regimen III: An Ultra Short Course Near-Universal Regimen

Our initial in vitro studies demonstrated that EMB and the experimental TB drug SQ109 were almost completely interchangeable even though they have slightly different mechanisms of action.^[15]^ This prompted us to replace EMB in PRS Regimen II with SQ109 to obtain PRS Regimen III (CFZ, SQ109, BDQ, and PZA).^[17]^ PRS Regimen III is a potential near universal regimen for treating TB, as it can be used to treat not only drug-sensitive TB but virtually all cases of drug-resistant TB, including MDRTB, since it contains three nonstandard drugs, and XDRTB, since it additionally does not contain either a fluoroquinolone or aminoglycoside.

To evaluate the efficacy of PRS Regimen III, we first determined the optimal in vivo doses by mapping the drug-dose efficacy response surface in the same way as we did for PRS Regimens I and II. The optimal doses of CFZ, SQ109, BDQ, and PZA in PRS Regimen III were 25, 25, 30, and 450 mg kg^−1^, respectively.

### 6.1. Early Bactericidal Activity of PRS Regimen III is Nearly Threefold That of the Standard Regimen

Before assessing the long-term efficacy of PRS Regimen III, we carried out a study of its early bactericidal activity (EBA) by determining in the BALB/c mouse model of pulmonary TB the average daily rate at which drug treatment reduces log CFU in the lung when the drugs are administered daily by gavage for 14 days (EBA_14_); the EBA_14_ is calculated simply by dividing the total reduction in log CFU from the first day of treatment (Day 1) to the end of the 14th day of treatment (Day 15) by 14. This assessment mirrors a test done in humans treated for TB where the EBA_14_ of drugs or drug combinations is determined by assaying CFU of Mtb in the patient’s sputum with 14 days of treatment. We compared the EBA_14_ of PRS Regimen III with that of PRS Regimen II and the Standard Regimen. Whereas the EBA_14_ for the Standard Regimen was 0.12 log CFU per day, the EBA_14_ determinations for PRS Regimens II and III in the same experiment were 0.33 and 0.34 log CFU per day, respectively, a rate of reduction almost threefold that of the Standard Regimen.^[17]^

### 6.2. PRS Regimen III Markedly Shortens Time to Lung Sterilization and Relapse-Free Cure

**Figure 7:**
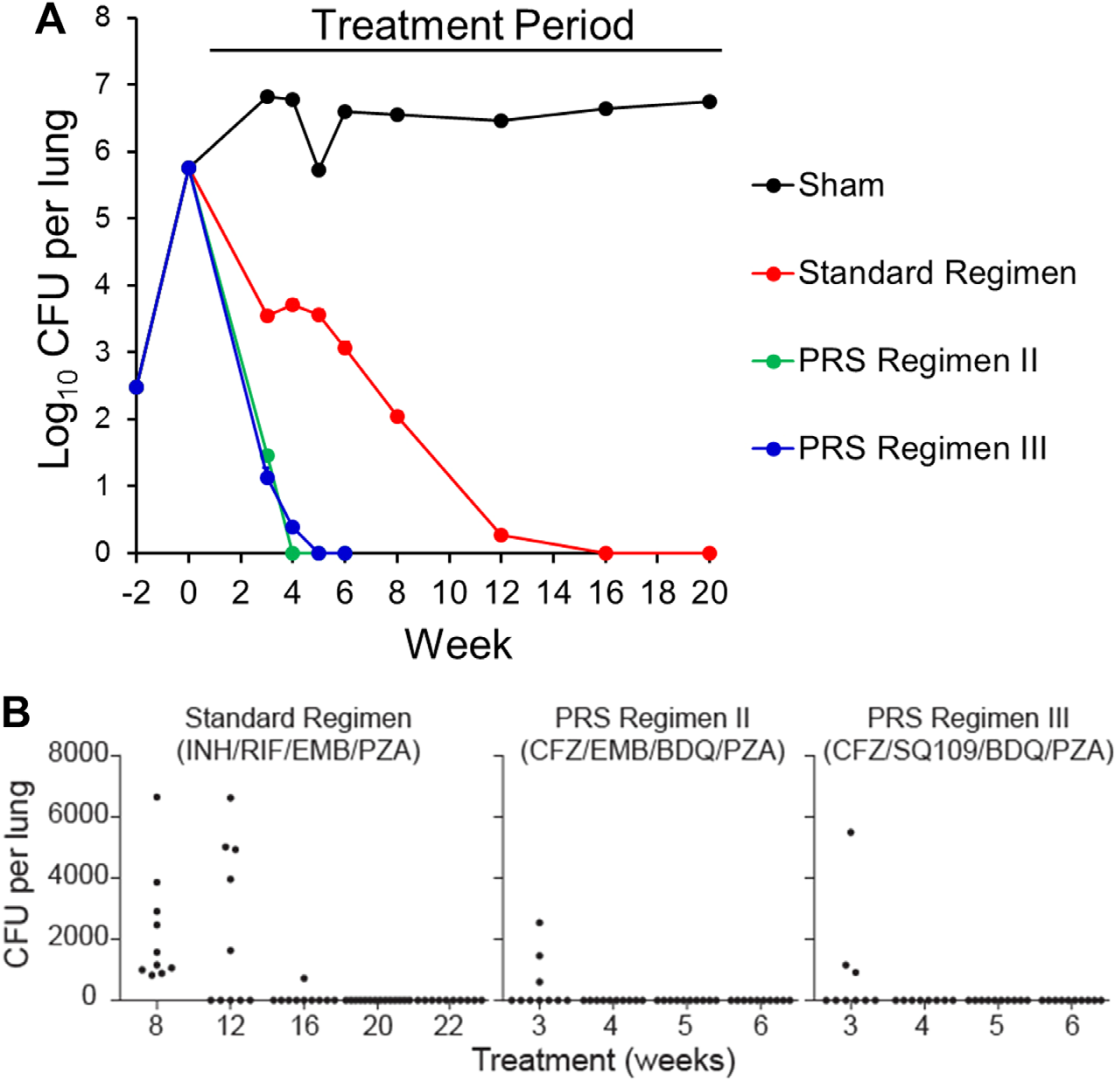
PRS Regimen III: long-term efficacy (time to lung sterilization) and relapse (time to relapse-free cure) study in BALB/c mice. A) Efficacy. Lung burden of Mtb in sham-treated mice and mice treated with the Standard Regimen, PRS Regimen II, or PRS Regimen III over the course of infection. For mice with zero CFU in the lungs, a CFU count of 1 was assigned for graphing purposes. B) Relapse. Total number of Mtb in the lung of each mouse was determined 3 months after treatment cessation. Relapse is defined as 1 or more CFU per lung. For all PRS Regimen II groups, PRS Regimen III groups at 5 and 6 weeks, and for the Standard Regimen groups at 8, 12, 16, and 22 weeks, *n* = 10 mice per group. For the PRS Regimen III group at 3 and 4 weeks, *n* = 9 and *n* = 8 mice per group, respectively, and for the Standard Regimen group at 20 weeks, *n* = 14 mice per group. (*p <* 0.0001, PRS Regimen III or PRS Regimen II versus Standard Regimen, log rank test). Adapted under the terms of the CC-BY License.^[17]^ Copyright 2018, The Authors.

Similar to PRS Regimen II, PRS Regimen III markedly reduced the time to lung sterilization and to relapse-free cure (**Figure 7**). In the same experiment in which the Standard Regimen required 20 weeks to achieve relapse-free cure, PRS Regimens II and III achieved relapse-free cure in 4 weeks, an 80% reduction in time.^[17]^

### 6.3. PRS Regimen III is as Effective in a Highly Susceptible Mouse Model As in a Conventional Mouse Model of Pulmonary TB

**Figure 8:**
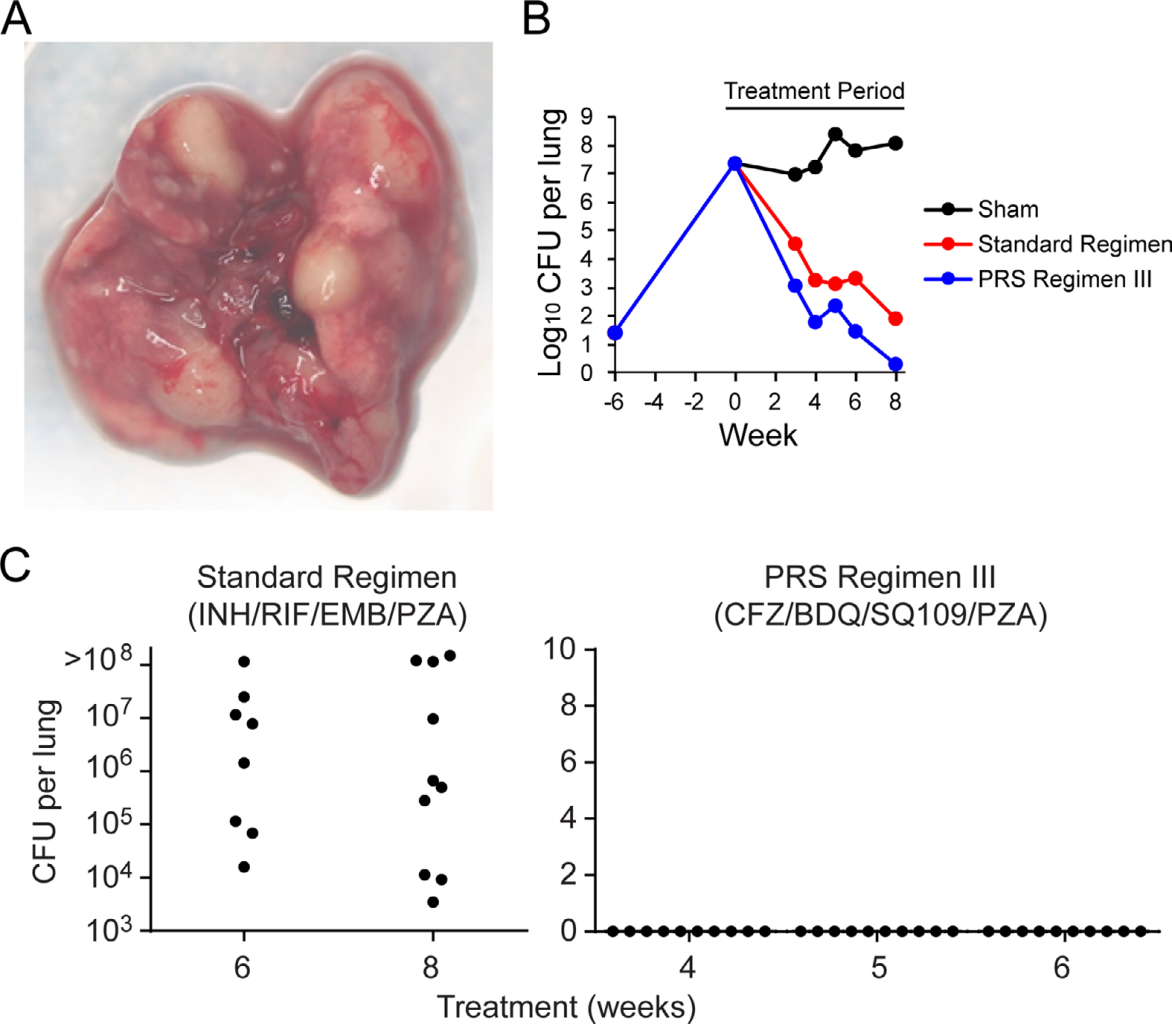
PRS Regimen III: treatment efficacy and time to relapse-free cure in C3HeB/FeJ mice. A) Pathology. Lung from Mtb-infected C3HeB/FeJ mouse showing massive granulomas. B) Treatment efficacy. Lung burden of Mtb over the course of infection after sham treatment or treatment with the Standard Regimen or PRS Regimen III. C) Relapse. Total number of Mtb in the lung of each mouse was determined 3 months after treatment cessation. Relapse is defined as 1 or more CFU per lung. For the Standard Regimen group at 8 weeks, and PRS Regimen III groups at 4 and 6 weeks, *n* = 10 mice per group. For Standard Regimen group at 6 week, *n* = 8 mice per group, and the PRS Regimen III group at 5 weeks, *n* = 9 mice per group. (*p <* 0.0001, PRS Regimen III vs Standard Regimen, log rank test). Reproduced under the terms of the CC-BY License.^[17]^ Copyright 2018, The Authors.

The BALB/c mouse model of pulmonary TB is the usual one used for TB drug studies. These mice develop granulomas in their lungs although not the very large caseating granulomas that occur in humans infected with Mtb. In contrast, C3HeB/FeJ mice develop massive caseating granulomas in their lungs more akin to the situation in humans.^[22]^ Some drugs have lower activity or less penetration into regions of caseous necrosis, and/or show disparate activity in the two mouse models of pulmonary TB,^[23–26]^ raising the concern that drug efficacy studies in the BALB/c mouse model may not be as predictive of drug efficacy in humans as studies in the C3HeB/FeJ mouse model, although the efficacy of the Standard Regimen is comparable in the two mouse models of pulmonary TB.^[27]^ In view of this concern, we explored the efficacy of PRS Regimen III in the C3HeB/FeJ mouse model in studies similar to those carried out in the BALB/c model. One difference is that we infected the C3HeB/FeJ mice by aerosol with a lower dose of Mtb, but then waited 6 weeks instead of 2 weeks for the Mtb to grow up in the mouse lungs prior to initiating treatment; the longer pretreatment period allowed the mice to develop massive caseating granulomas. By 6 weeks after infection, Mtb CFU in the lung had reached a very high level of 7.4 log CFU, ≈1.6 logs higher than in BALB/c mice at the start of treatment; as predicted, they had very large caseating granulomas. Despite the higher lung burden of Mtb at the onset of treatment and the striking pathology in the C3HeB/FeJ mice, PRS Regimen III rapidly reduced bacterial burden in the lungs of the C3HeB/FeJ mice to provide a 100% relapse-free cure after 4 weeks treatment, the same amount of time required in the BALB/c mice (**Figure 8**). By contrast, 0% of mice treated with the Standard Regimen achieved relapse-free cure at 6 or 8 weeks after the start of treatment. Hence, PRS Regimen III was as effective at achieving relapse-free cure in C3HeB/FeJ mice with massive caseating granulomas as in BALB/c mice.

## 7. PRS Regimens IV and V: Ultra Short-Course Near-Universal Regimens Comprising Approved Drugs

In a second series of studies, we expanded the pool of TB drugs studied to 15 by adding the drug delamanid (DLM) to the original list of drugs.^[18]^ In vitro studies employing the Mtb-infected macrophage model identified promising new four-drug combinations. We screened 14 such combinations in the BALB/c mouse model of pulmonary TB, using the highest well-tolerated dose of each drug in each drug regimen since in previous drug-dose efficacy response mapping studies, the group treated with the high dose of each drug in the combination had among the lowest CFU burdens in the lung even though the optimal drug ratios typically involved a lower dose of some of the drugs in the regimen. On the basis of this screening study, we identified two additional highly potent drug combinations: PRS Regimen IV comprising CFZ, BDQ, PZA, and A/C, and PRS Regimen V comprising CFZ, BDQ, PZA, and DLM. Interestingly, both combinations included three of the drugs common to PRS Regimens II and III: CFZ, BDQ, and PZA, suggesting that these three drugs provide an unusually potent combination against TB. As each of these regimens comprised only approved drugs, contained three nonstandard TB drugs, and did not contain a fluoroquinolone or aminoglycoside, they constituted rapid-acting near-universal drug regimens for treating TB comprised of drugs currently available to clinicians for treating TB. In view of the consistent presence of CFZ, BDQ, and PZA in each of the most potent drug regimens identified in two series of studies, we designated these three drugs as PRS Regimen VI.

**Figure 9:**
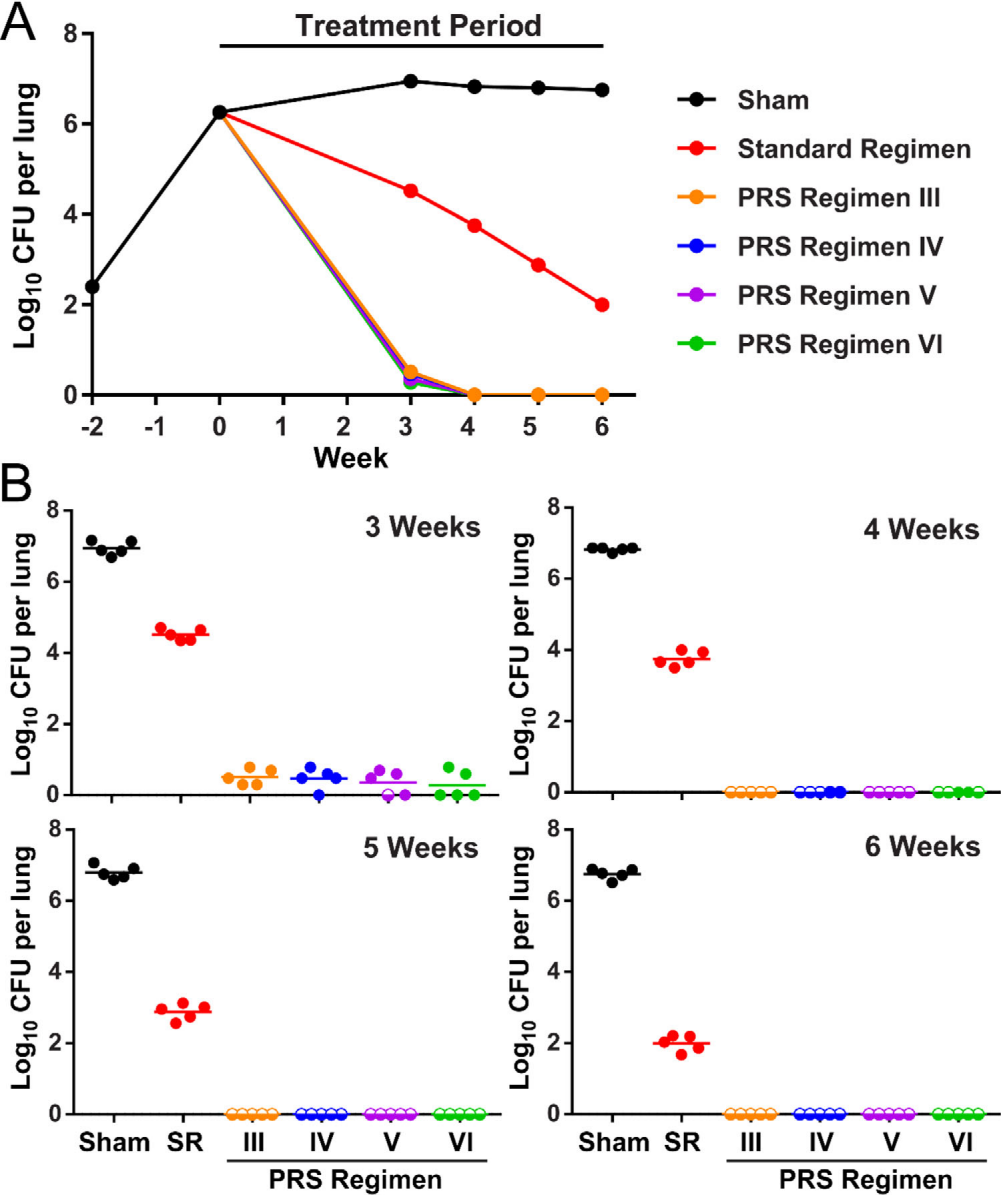
PRS Regimens IV–VI: Treatment efficacy in BALB/c mice. A) Time course of the bacterial burden in the lung over the infection and treatment period. B) Lung burden of Mtb after treatment 5 days per week for 3, 4, 5, and 6 weeks in sham-treated mice or mice treated with the Standard Regimen (SR) or one of the PRS Regimens (III–VI). Mice with zero CFU in the lungs are plotted as log 0 CFU on the scale and indicated by a semi-open circle symbol. Reproduced under the terms of the CC-BY License.^[18]^ Copyright 2019, The Authors.

Mapping of the drug-dose efficacy response surface indicated that the optimal doses of CFZ, BDQ, PZA, and A/C in PRS Regimen IV were 25, 37, 50, and 66.7/16.7 mg kg^−1^, respectively, and the optimal doses of CFZ, BDQ, PZA, and DLM in PRS Regimen V were 25, 40, 185, and 0.83 mg kg^−1^, respectively. Extrapolating from these studies, the optimal doses of CFZ, BDQ, and PZA in PRS Regimen VI were 25, 40, and 185 mg kg^−1^, respectively. Using these optimal doses, we determined that the EBA_14_ for PRS Regimens IV, V, and VI were 0.37, 0.36, and 0.36 log CFU per day, respectively, versus 0.14 log CFU per day for the Standard Regimen in the same experiment. As with PRS Regimens II and III, the EBA_14_ for these regimens was almost threefold greater than that of the Standard Regimen. Efficacy studies showed that PRS Regimens IV–VI reduced CFU in the lungs of Mtb-infected BALB/c mice at the same rate as PRS Regimen III (**Figure 9**). PRS Regimens III, V, and VI achieved 100% relapse-free cure in 3 weeks (10 mice per group); PRS Regimen IV achieved 78% relapse-free cure at 3 weeks (9 mice per group), 90% relapse-free cure at 4 weeks (10 mice per group), and 100% relapse-free cure at 5 weeks (10 mice per group).

## 8. Conclusions

AI-enabled PRS methodology in conjunction with an in vitro Mtb-infected macrophage cell culture assay amenable to high-throughput screening allows rapid identification of highly effective drug regimens suitable for further in vivo screening in a mouse model of pulmonary TB. The same AI-enabled PRS platform allows optimization of the in vivo doses of each drug in a regimen for definitive testing of the regimen’s efficacy in sterilizing the lung and achieving relapse-free cure in a mouse model of pulmonary TB. Using these methods, we have identified several PRS regimens that are dramatically more effective than the Standard Regimen, achieving relapse-free cure in as little as 3 or 4-weeks treatment, compared with 16–20 weeks required for the Standard Regimen—an ≈80% reduction in time. We anticipate that applying this methodology to new drugs in development will allow further shortening of the time required to achieve relapse-free cure and will be a game changer in the treatment of TB.

## References

[1] WHO global tuberculosis report 2017, https://www.who.int/tb/publications/global_report/en/ Accessed April 2019.

[2] Ding X., Sanchez D. J., Shahangian A., Al-Shyoukh I., Cheng G., Ho C. M., *Int. J. Nanomedicine* 2012, 7, 2281. 22654513 10.2147/IJN.S27540 PMC3363951

[3] Weiss A., Berndsen R. H., Ding X., Ho C. M., Dyson P. J., van den Bergh H., Griffioen A. W., Nowak-Sliwinska P., *Sci. Rep.* 2015, 5, 14508. 26416286 10.1038/srep14508 PMC4586442

[4] Wang H., Lee D. K., Chen K. Y., Chen J. Y., Zhang K., Silva A., Ho C. M., Ho D., *ACS Nano* 2015, 9, 3332. 25689511 10.1021/acsnano.5b00638

[5] Nowak-Sliwinska P., Weiss A., Ding X., Dyson P. J., van den Bergh H., Griffioen A. W., Ho C. M., *Nat. Protoc.* 2016, 11, 302. 26766116 10.1038/nprot.2016.017

[6] Rashid M. B. M. A., Toh T. B., Silva A., Nurrul Abdullah L., Ho C.-M., Ho D., Chow E. K.-H., *J. Lab. Autom.* 2015, 20, 423. 25824204 10.1177/2211068215579612

[7] Weiss A., Ding X., van Beijnum X. J. R., Wong I., Wong T. J., Berndsen R. H., Dormond O., Dallinga M., Shen L., Schlingemann R. O., Pili R., Ho C.-M., Dyson P. J., van den Bergh H., Griffioen A. W., Nowak-Sliwinska P., *Angiogenesis* 2015, 18, 233. 25824484 10.1007/s10456-015-9462-9 PMC4473022

[8] Chen Y.-T., Goudar V. S., Wu R.-G., Hsieh H.-Y., Yang C.-S., Chang H.-Y., Lee G.-B., Ho C.-M., Tseng F.-G., *RSC Adv.* 2016, 6, 44425.

[9] Liu Q., Zhang C., Ding X., Deng H., Zhang D., Cui W., Xu H., Wang Y., Xu W., Lv L., Zhang H., He Y., Wu Q., Szyf M., Ho C.-M., Zhu J. *Sci. Rep.* 2015, 5, 11464. 26088171 10.1038/srep11464 PMC5155572

[10] Rashid M. B.M. A., Toh T. B., Hooi L., Silva A., Zhang Y., Tan P. F., Teh A. L., Karnani N., Jha S., Ho C.-M., Chng W. J., Ho D., Chow E. K., *Sci. Transl. Med.* 2018, 10, eaan0941. 30089632 10.1126/scitranslmed.aan0941

[11] Lee D.-K., Chang V. Y., Kee T., Ho C.-M., Ho D. *SLAS Technol.* 2017, 22, 276. 27920397 10.1177/2211068216681979

[12] Ding X., Njus Z., Kong T., Su W., Ho C.-M., Pandey S., *Sci. Adv.* 2017, 3, eaao1254. 28983514 10.1126/sciadv.aao1254 PMC5627981

[13] Zarrinpar A., Lee D. K., Silva A., Datta N., Kee T., Eriksen C., Weigle K., Agopian V., Kaldas F., Farmer D., Wang S. E., Busuttil R., Ho C.-M., Ho D. *Sci. Transl. Med.* 2016, 8, 333ra49. 10.1126/scitranslmed.aac5954 27053773

[14] Ma Z., Lienhardt C., McIlleron H., Nunn A. J., Wang X., *Lancet* 2010, 375, 2100. 20488518 10.1016/S0140-6736(10)60359-9

[15] Silva A., Lee B. Y., Clemens D. L., Kee T., Ding X., Ho C. M., Horwitz M. A., *Proc. Natl. Acad. Sci. U.S.A.* 2016, 113, E2172. 27035987 10.1073/pnas.1600812113 PMC4839402

[16] Lee B. Y., Clemens D. L., Silva A., Dillon B. J., Masleša-Galić S., Nava S., Ding X., Ho C. M., Horwitz M. A., *Nat. Commun.* 2017, 8, 14183. 28117835 10.1038/ncomms14183 PMC5287291

[17] Lee B. Y., Clemens D. L., Silva A., Dillon B. J., Masleša-Galić S., Nava S., Ho,M. A.Horwitz C. M., *PLoS One* 2018, 13, e0207469. 30427938 10.1371/journal.pone.0207469 PMC6235396

[18] Clemens D. L., Lee B. Y., Silva A., Dillon B. J., Masleša-Galić S., Nava S., Ding X., Ho C. M., Horwitz M. A., *PLoS One* 2019, 14, e0215607. 31075149 10.1371/journal.pone.0215607 PMC6510528

[19] Lee B. Y., Clemens D. L., Horwitz M. A., *Mol. Microbiol.* 2008, 68, 1047. 18363792 10.1111/j.1365-2958.2008.06214.x PMC3591484

[20] Rosenthal I. M., Zhang M., Williams K. N., Peloquin C. A., Tyagi S., Vernon A. A., Bishai1 W. R., Chaisson R. E., Grosset J. H., Nuermberger E. L., *PLoS Med.* 2007 *4*, e344. 10.1371/journal.pmed.0040344 PMC2140085 18092886

[21] Tyagi S., Ammerman N. C., Li S.-Y., Adamson J., Converse P. J., Swanson R. V., Almeida D. V., Grosset J. H. *Proc. Natl. Acad. Sci. U.S.A.* 2015, 112, 869, 10.1073/pnas.1416951112 25561537 PMC4311815

[22] Kramnik I., *Curr. Top. Microbiol. Immunol.* 2008, 321, 123. 18727490 10.1007/978-3-540-75203-5_6

[23] Lanoix J. P., Betoudji F., Nuermberger E., *Antimicrob. Agents Chemother.* 2016, 60, 1091. 26643352 10.1128/AAC.02637-15 PMC4750691

[24] Irwin S. M., Prideaux B., Lyon E. R., Zimmerman M. D., Brooks E. J., Schrupp C. A., Chen C., Reichlen M. J., Asay B. C., Voskuil M. I., Nuermberger E. L., Andries K., Lyons M. A., Dartois V., Lenaerts A. J., *ACS Infect. Dis.* 2016, 2, 251. 27227164 10.1021/acsinfecdis.5b00127 PMC4874602

[25] Lanoix J. P., Lenaerts A. J., Nuermberger E. L., *Dis. Models Mech.* 2015, 8, 603. 10.1242/dmm.019513 PMC4457036 26035868

[26] Irwin S. M., Gruppo V., Brooks E., Gilliland J., Scherman M., Reichlen M. J., Leistikow R., Kramnik I., Nuermberger E. L., Voskuil M. I., Lenaerts A. J., *Antimicrob. Agents Chemother.* 2014, 58, 4026. 24798275 10.1128/AAC.02565-14 PMC4068578

[27] Li S. Y., Irwin S. M., Converse P. J., Mdluli K. E., Lenaerts A. J., Nuermberger E. L., *Antimicrob. Agents Chemother.* 2015, 59, 4026. 25918146 10.1128/AAC.00105-15 PMC4468727

